# Unique Chemistry, Intake, and Metabolism of Polyamines in the Central Nervous System (CNS) and Its Body

**DOI:** 10.3390/biom12040501

**Published:** 2022-03-25

**Authors:** Julian Rieck, Serguei N. Skatchkov, Christian Derst, Misty J. Eaton, Rüdiger W. Veh

**Affiliations:** 1 Institut für Zell- und Neurobiologie, Centrum 2, Charité—Universitätsmedizin Berlin, Charitéplatz 1, D-10117 Berlin, Germany; julian.rieck@charite.de; 2 Department of Physiology, Universidad Central del Caribe, Bayamón, PR 00956, USA; 3 Department of Biochemistry, Universidad Central del Caribe, Bayamón, PR 00956, USA; misty.eaton@uccaribe.edu; 4 Institut für Integrative Neuroanatomie, Centrum 2, Charité—Universitätsmedizin Berlin, Charitéplatz 1, D-10117 Berlin, Germany; christian.derst@t-online.de

**Keywords:** polyamines, CNS, astrocytes, neurons, glial cells, spermidine, spermine, agmatine, nutrition, transport

## Abstract

Polyamines (PAs) are small, versatile molecules with two or more nitrogen-containing positively charged groups and provide widespread biological functions. Most of these aspects are well known and covered by quite a number of excellent surveys. Here, the present review includes novel aspects and questions: (1) It summarizes the role of most natural and some important synthetic PAs. (2) It depicts PA uptake from nutrition and bacterial production in the intestinal system following loss of PAs via defecation. (3) It highlights the discrepancy between the high concentrations of PAs in the gut lumen and their low concentration in the blood plasma and cerebrospinal fluid, while concentrations in cellular cytoplasm are much higher. (4) The present review provides a novel and complete scheme for the biosynthesis of Pas, including glycine, glutamate, proline and others as PA precursors, and provides a hypothesis that the agmatine pathway may rescue putrescine production when ODC knockout seems to be lethal (solving the apparent contradiction in the literature). (5) It summarizes novel data on PA transport in brain glial cells explaining why these cells but not neurons preferentially accumulate PAs. (6) Finally, it provides a novel and complete scheme for PA interconversion, including hypusine, putreanine, and GABA (unique gliotransmitter) as end-products. Altogether, this review can serve as an updated contribution to understanding the PA mystery.

## 1. Introduction

The term polyamines (PAs) comprises small molecular compounds with two or more nitrogen-containing, positively charged groups, such as ammonium or guanidinium residues (Table 1). PAs may be derived from natural or artificial sources. PAs and their metabolites may be encountered throughout all kingdoms of life [1]. The important PAs in mammalian cells are putrescine (PUT), spermidine (SPD), and spermine (SPM), which also are present in all eukaryotic cells. Some prokaryotes may lack the ability to synthesize SPM [2], but thermophiles [3], especially, show a wide variety of other PAs (Table 1).

Biological functions are widespread, including simply buffering acidic compartments [4,5], stabilizing or condensing nucleic acids [6,7], promoting homology-directed DNA repair [8], protecting from oxidative damage [9,10], and increasing longevity [11,12,13], and are covered in a separate article of this special issue.

Historically, PAs had been discovered during the seventeenth century, when crystals appeared in samples of human semen left to cool [14]. A century later, these crystals were identified as an organic phosphate [15]. After another 100 years, the organic base was identified [16] and subsequently called “spermine” [16,17]. Later, PA biosynthesis, interconversions, and basic biological functions were established by E. Agostinelli, U. Bachrach, R. Casero, T. Eisenberg, G. Gilad, K. Igarashi, J. Jänne, F. Madeo, G. Park, M. Pegg, C. W. Porter, M. Rosenheim, H. Tabor, C. W. Tabor, H. Wallace, and other scientists (alphabetical order; for reviews see [18,19,20,21]). The interest in PAs has grown steadily, with 1 paper published on PA-research from 1951 to 1960, 2259 papers from 1981 to 1990, and 4113 papers from 2011 to 2020.

The existing literature on PAs is abundant but sometimes vague. The present review focuses on chemistry, nutritional uptake, and metabolism of PAs in the mammalian central nervous system (CNS) and may serve as a framework for an increased molecular understanding of PA homeostasis.

## 2. Chemistry of Polyamines

Most PAs consist of at least two amino or guanidinium groups that are positively charged and separated by a carbon backbone of varying length. In contrast to other cations, such as Mg^2+^ or Ca^2+^, PAs feature at least two charged groups connected by a flexible carbon chain. This adds hydrophobic effects [22,23] to the electrostatic interactions of the amino or guanidine groups. SPM and SPD are the most abundant PAs in mammals. PUT is the common precursor but is usually only present in low concentrations [3] or is even absent in some parasitic organisms [24]. Chemically, agmatine (AGM) also belongs to the PA family. This biogenic amine, however, exerts largely separate functions in mammalian tissues as compared to the other PAs [25,26], but may serve as a precursor for PUT [27,28,29].

## 3. Intake of Polyamines from Nutrition

**Figure 1:**
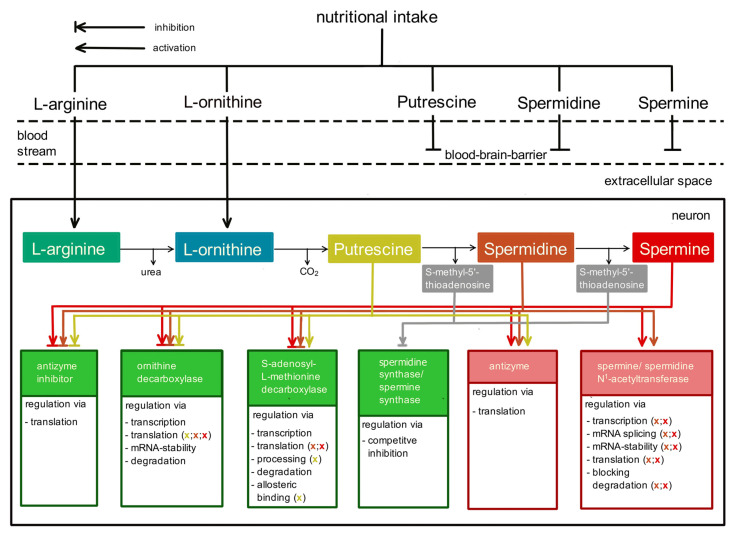
Schematic representation of nutritional intake and regulated biosynthesis to maintain polyamine homeostasis in the body. Nutritional intake into the blood stream includes arginine and orhithine in addition to the polyamines (PAs) putrescine, spermidine, and spermine. PAs are taken up mostly in the small intestine and delivered to the blood stream, but they cannot pass the blood–brain barrier. However, arginine and ornithine can and will represent starting materials of the regulated biosynthesis of PAs in the central nervous system. Inside the neuron, PA concentrations are tightly controlled by at least six proteins, represented as boxes in the bottom row: antizyme inhibitor, ornithine decarboxylase, S-adenosylmethionine decarboxylase, Spd/Spm-synthase, antizyme, and Spm/Spd-N^1^-acetyltransferase. Arrows or blocked arrows indicate which target proteins (green: increase PAs; red: decrease PAs) are modulated by a given PA. The colour of the (x) indicates which PA is involved.

Polyamines (PAs) may be derived from alimentary sources or by biosynthesis. In the mammalian gut lumen, the predominant share of PAs stems from food intake [30], whereas a variable quantity may be produced by large intestine microbiota [31]. The intestinal flora in most mammals synthesizes mainly PUT, whereas its amount depends on the composition of the diet [31]. Food sources with relevant PA content include cheese, nuts, mushrooms, tea, fruit, vegetables, mollusks, and other meat products [32,33,34,35]. With respect to PA synthesis by gut microbiota, an arginine rich diet is favorable [31]. PAs in the gut lumen are absorbed predominantly by the duodenal and jejunal mucosa [36,37] and subsequently transferred [31] into the bloodstream (Figure 1). The estimated average of alimentary intake of PAs into the gut varies between 250 to 550 µmol/d, depending on the geographical region and associated food patterns [32]. In detail, PUT contributes 197 µmol/d, SPD with 74 µmol/d, and SPM with 46 µmol/d [34] to the daily PA intake.

PAs in the gut lumen may reach almost millimolar concentrations after a meal and disappear rapid and completely [37], whereby the luminal PA content returns to the fasting level in about 120 min [30]. PUT is metabolized almost completely inside the enterocytes. Plasma levels of SPD and SPM show only mild (up to 20 µM) increases after a meal [30]. Most likely, PA uptake in peripheral tissues keeps the plasma concentrations low (Table 2).

Loss of PAs from the body may be due to micturition and defecation. Concentrations in urine are very low (Table 2) and PA loss via diuresis is negligible. Estimated concentrations in feces are about 800 µM for PUT, about 40 µM for SPD, and 20 µM for SPM [36]. Considering fecal density of 1.09 kg/L and 155 g as an average daily amount of stool, loss of PAs in feces amounts to about 123 µmoles PUT, 5.9 µmoles SPD, and 2.9 µmoles SPM per day. Altogether, there is roughly a nutritional daily net intake into the body of 74 µmoles PUT, 68 µmoles SPD and 43 µmoles SPM. Actually, the uptake of PUT may be much higher, as gut microbiota can produce considerable amounts of PUT from arginine [31].

The brain apparently is excluded from taking up PAs from the plasma (Figure 1), as the blood–brain barrier (BBB) seems to be completely impermeable to PAs [38,39] (this special issue). Consequently, the brain depends on different sources to obtain the essential PAs. Most likely, arginine (Figure 1), which crosses the blood–brain-barrier via the CAT1 transporter [40], provides the necessary material for PA biosynthesis in brain.

## 4. Biosynthesis of Polyamines

**Figure 2:**
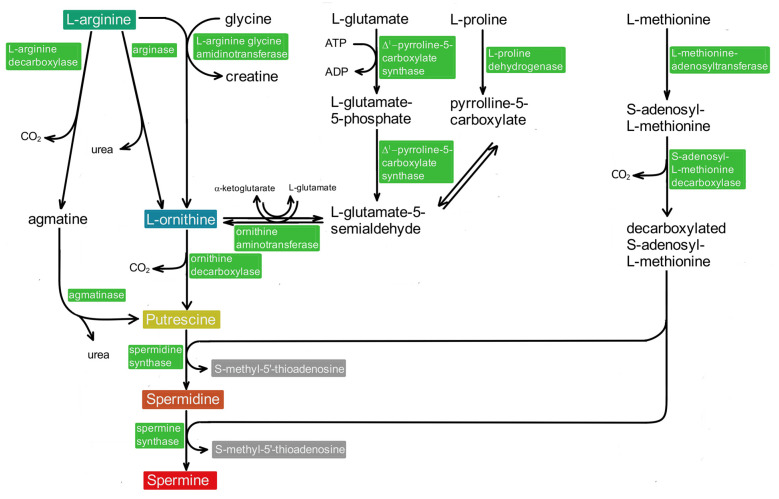
Schematic representation of polyamine biosynthesis. The most important biosynthetic pathway begins with the action of arginase on arginine, forming ornithine, and its subsequent decarboxylation provides putrescine (PUT). PUT may also be obtained from arginine via decarboxylation to agmatine and subsequent action of agmatinase (**left** column). In addition, ornithine may be obtained from arginine and glycine via arginine-glycine-amidinotransferase. Glutamate and proline provide additional sources (**middle** columns). The additional carbon chains of spermidine and spermine are derived from methionine (**right** column).

**Figure 3:**
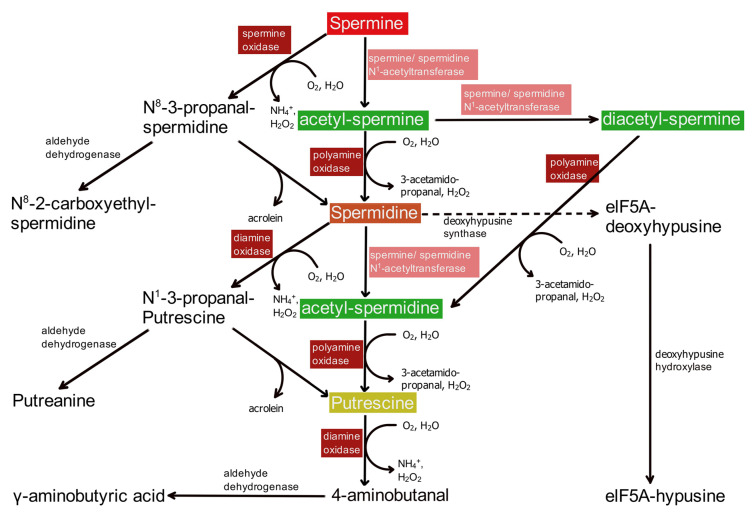
Schematic representation of polyamine catabolism and conversions. In the first step, the classical degradation pathway for spermine and spermidine involves N-acetylation via the corresponding N-acetyl transferase. N-acetylated PAs are oxidized by peroxisomal polyamine oxidase, yielding spermidine or putrescine, respectively. Alternately, spermine may be directly oxidized by spermine oxidase to N^8^-3-propyl-spermidine (**upper left** side), which spontaneously splits off acrolein and thus is converted to spermidine. This molecule, instead of N-acetylation, may be oxidized by diamine oxidase to N^1^-3-propyl-putrescine (**lower left** side), which again under loss of acrolein forms putrescine. There are two side pathways. Spermidine may be attached to a lysine side chain of a nascent protein, which subsequently is hydroxylated to the functional elF5A-hypusine transcription factor (**right** side). In a separate pathway, N^1^-3-propyl-putrescine, the product of the oxidation of N-acetyl-spermidine by diamine oxidase, is reduced to putreanine.

When the brain is excluded from nutritional PA sources, homeostasis (Figure 1) depends on metabolism. With respect to the biological importance of PAs, it is plausible that their intracellular concentration is tightly controlled at several levels. This task is fulfilled by regulating biosynthesis (Figure 2) as well as degradation (Figure 3). The amino acids L-arginine, L-ornithine, glycine, L-proline, L-glutamate, and L-methionine [41,42,43] are effective sources. Biosynthesis predominantly follows two different pathways, both producing PUT as starting material for the following reactions (Figure 2).

### 4.1. The Ornithine Decarboxylase Pathway

In the “classical” ornithine decarboxylase (ODC) pathway (Figure 2), arginine is split by arginase, yielding ornithine and urea [42,44]. Alternately [42,45], ornithine may be obtained from arginine via a reaction with glycine, from glutamate, or from proline (Figure 2).

PUT, the starting product for the biosynthesis of SPD and SPM, is generated from ornithine via decarboxylation by ODC. This is the rate-limiting step and, consequently, mammalian PA synthesis is predominantly controlled here (Figure 1). The amount of ODC enzyme is increased by low levels, while its degradation is enhanced by high levels of PAs. ODC function is further subjected to regulation by ODC antizyme and, in addition, by an antizyme inhibitor protein [46,47,48]; for review see [3]), highlighting the intricate control mechanisms that guarantee intracellular PA homeostasis.

In the next biosynthesis step (Figure 2), spermidine synthase, an aminopropyl transferase, adds an aminopropyl group derived from decarboxylated S-adenosylmethionine (dcAdoMet) to PUT, thereby forming SPD. Subsequently, SPD is elongated the same way by another aminopropyl group, now via spermine synthase, to form SPM. Both aminopropyl transferases are subject to negative feedback control (Figure 1) via their reaction product S-methyladenosine [49]. Since a steady supply of dcAdoMet is crucial for de novo synthesis of SPD and SPM, its formation also represents a rate-limiting step in PA biosynthesis [3,44,46]. Consequently, the corresponding enzyme, S-adenosylmethionine decarboxylase (AdoMetDC), also is tightly regulated at several levels (Figure 1; [1,47]; for review see [3]).

### 4.2. The Agmatine Pathway

The second pathway starts with enzymatic cleavage of arginine (Figure 2) by arginine decarboxylase into agmatine (AGM). Subsequently, AGM is split into PUT and urea by agmatinase [50,51,52,53]. It still remains somewhat unclear whether the pathway for the biosynthesis of AGM, an important neurotransmitter, plays a major role for the production of PUT and other PAs.

The fact is that mouse knockouts of ODC, which are completely devoid of PA biosynthesis via the ODC pathway, do not survive early stages of embryonic development [54]. Blocking PA biosynthesis via difluoromethylornithine, an ODC inhibitor, stops glial cell proliferation [53] and causes developmental arrest of the embryo in mouse [55], rat [56,57], rabbit [58], hamster [59], and mink [60]. These data suggest that at least in early stages of embryonic development, the AGM pathway is not sufficient to overcome the absence of ODC for PA biosynthesis.

When, however, biosynthesis of PUT is blocked via antisense oligonucleotides against ODC, the AGM pathway may be sufficient to rescue PA biosynthesis [51]. In addition, the fact that the AGM pathway is widely distributed also in peripheral tissues supports a general role of AGM in PA biosynthesis [29,61]. Both apparently contradictory facts could be explained by assuming that the AGM pathway is fully developed only later in life.

## 5. Concentrations and Transport of Polyamines within the Brain

Tissue concentrations strongly differ among individual PAs (Table 2). When focusing on SPM, it appears rather surprising that the intracellular concentration is very high (about 1 mm), whereas it is very low in plasma/serum (50 nM or below) and in cerebrospinal fluid (CSF; about 100 nm). Such a steep gradient of PA concentrations between blood plasma and CSF, extracellular brain space, cytoplasm, and total PA content in cells requires explanation. Most likely, the systems use different transporters [62].

The fact that PAs in the brain cannot be acquired from the blood stream [38,39] (this special issue) and consequently must be synthesized in the CNS itself (Figure 1) leads to the question of where this biosynthesis takes place, in neurons or in glial cells.

Very likely arginine, which crosses the blood–brain-barrier via the CAT1 transporter [40], represents the starting material. Arginine may be converted to ornithine by arginase and subsequently to PUT by ODC. Alternately, PUT may be derived from AGM via agmatinase. Arginase, ODC, and agmatinase are found in neurons [63,64,65]. The presence of any protein inside a cell, however, contains no information on its activity. Thus, ODC in the adult human brain is predominantly associated with its antizyme protein [64], resulting in low or absent enzymatic activity.

**Figure 4:**
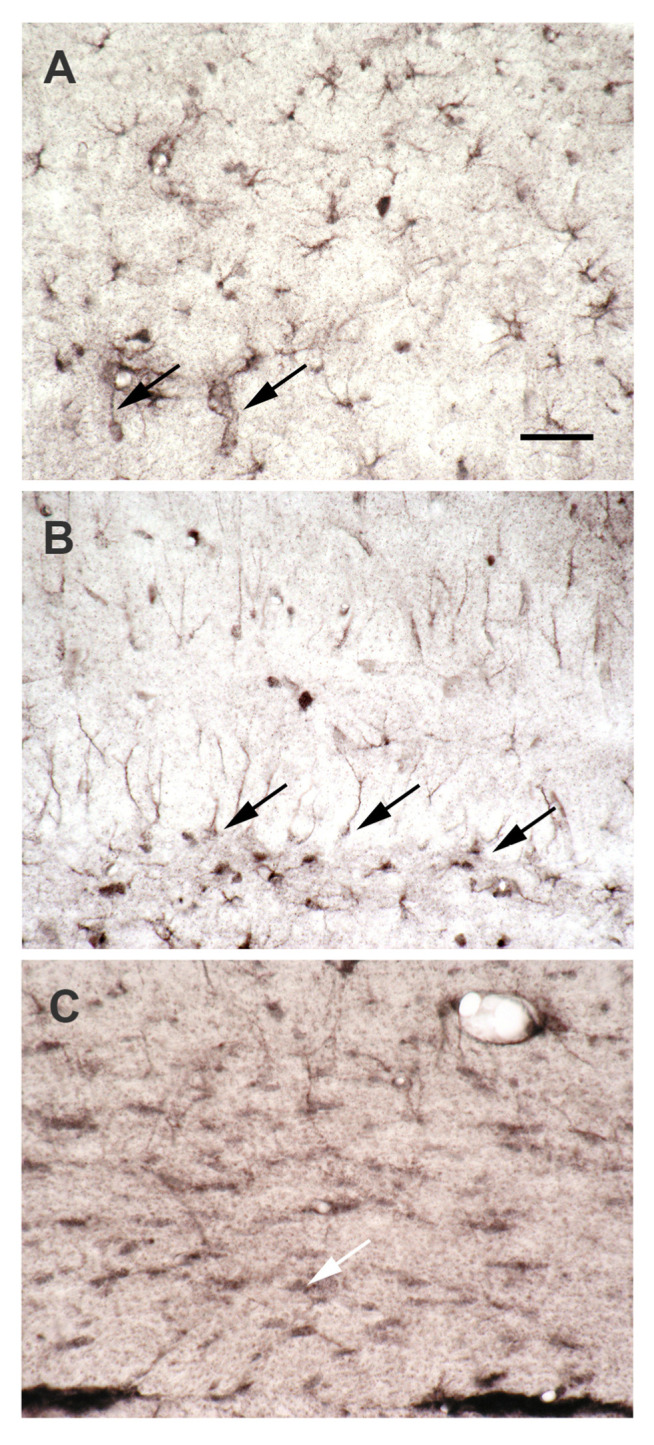
Spermidine/spermine immunoreactivity is predominantly localized in astrocytes, not in neurons. Coronal sections of rat hippocampus after immunocytochemical visualization of SPD/SPM. The antibody (raised in the author’s lab [66]) does not differentiate between tissue-bound spermidine and spermine. Thus, these two polyamines cannot be visualized separately. (**A**) Spd/spm-immunoreactivity in the CA1 region of the hippocampus is largely restricted to astrocytes. Some of their processes extend to capillaries (left arrow), forming endfeet there. Note the strong staining of the capillary walls (right arrow). Whether this staining is due to labeled astrocyte endfeet or to an immunopositive endothelium cannot be decided here. (**B**) In the dentate gyrus spd/spm-immunoreactivity of astrocytes displays a very different appearance. Many cell bodies are found at the lower border of the granule cell layer (arrows), with rather straight processes extending to the molecular layer. Other astrocytes with a similar morphology are found in more superficial regions of the dentate gyrus. In contrast, the bottom of the photograph presents the hilar area, where astrocytes show their usual appearance. (**C**) Among immunoreactive astrocytes, the corpus callosum also displays spd/spm-positive oligodendrocytes (white arrow). Taken from Höhlig et al., this special issue. Bar in (**A**) indicates 50 µm in (**A**–**C**).

**Figure 5:**
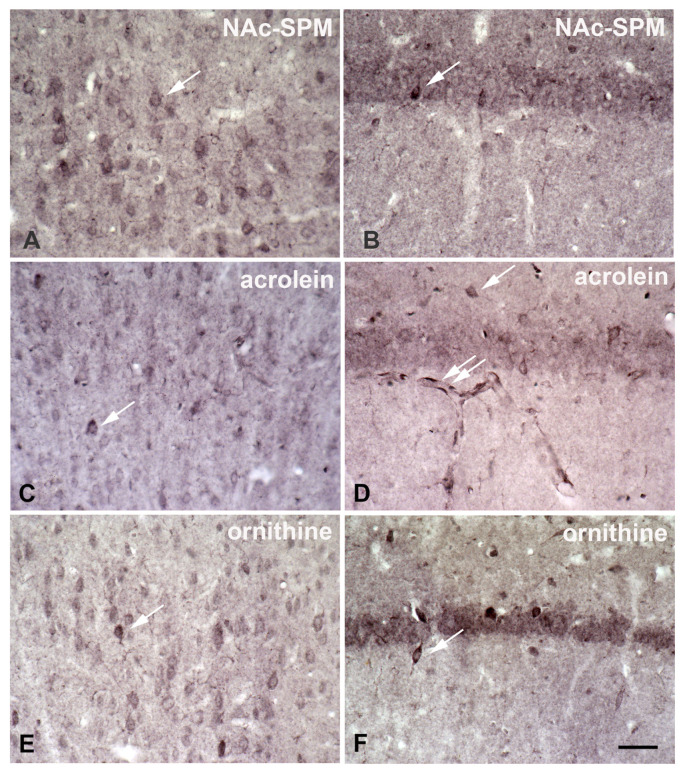
Immunocytochemical visualization of some components of polyamine metabolism. Coronal sections of rat cortex (**A**,**C**,**E**) and hippocampus (**B**,**D**,**F**) after immunocytochemical visualization of (1) N-acetylspermine, (2) acrolein, and (3) ornithine display staining predominantly in neurons. Antibodies had been raised in the author’s laboratory (1, 3) as described earlier [66] or were obtained from commercial sources (2, rabbit anti-acrolein; LS-C63521, MoBiTec, Göttingen, Germany). All control sections were negative. Surprisingly, immunoreactivity is more pronounced in interneurons (single arrows in all images) as compared to adjacent neurons in all sections. This indicates that there may be considerable differences between separate classes of neurons. Note the strong acrolein-immunoreactivity in capillary walls ((**D**), double arrow). Bar in (**F**) indicates 50 µm in all images. Taken from Höhlig et al., this special issue.

SPD/SPM immunoreactivity (Figure 4) is much more prominent in astrocytes as compared to neurons [66], which is surprising, because the synthesizing enzyme, ODC, appears to be restricted to neurons [64,67]. The data suggest that in the brain PAs may be primarily synthesized in neurons. This idea is strongly supported by the massive expression of SPD-synthase immunoreactivity exclusively in neurons [68]. PA degradation may occur in neurons and astrocytes, as SSAT [69], N-acetylspermine, and acrolein (Figure 5; from [70] this special issue and Figure S1) are localized in both cell types. PAs directly after N-acetylation can leave the cell [71] and are taken up by astrocytes. The capacity of this uptake system is enormous, allowing astrocytes to reach above 1 mM internal SPM after one hour (recalculation based on the data of [72]). Not all neurons, however, release all their intracellular Pas, as conspicuous neuronal SPD/SPM-like immunoreactivity is distributed in a regional- specific manner throughout the brain [73]. The biological meaning of separate synthesis and storage appears unclear at present. Novel data, however, suggest that there is an intense exchange of PAs during astrocytes and neurons. Thus, in the retina during daytime, PA-immunoreactivity is strongly enhanced in photoreceptor terminals, while at night, reactivity in Müller cells was predominant [74]. This indicates that the exchange of PAs between neurons and astrocytes may depend on respective activities.

The total concentration of intracellular SPM in different cells (Table 2) is estimated (presumably to high, see Table 2) to about 3–10 mM [4,75] and most is bound to negatively charged molecules, such as DNA, RNA, phospholipids, acidic proteins, and others. In contrast, the concentration of free SPM as deduced from functional and biochemical tests is much smaller [74,76,77,78]. Unfortunately, this fact often is misunderstood. When SPM is largely bound to a number of macromolecules, this does not mean that it is unavailable for interactions with others. Binding between molecules always is an equilibrium process. Thus, availability of SPM does not depend on the amount, which is bound to other molecules, but only on the affinity of these interactions. When these are low (as they are) and affinities to ion channels or receptors are high (as they are), SPM moves very fast (below milliseconds) to such other binding sites. Therefore, the amount of free as compared to total PAs inside a cell is rather unimportant.

**Figure 6:**
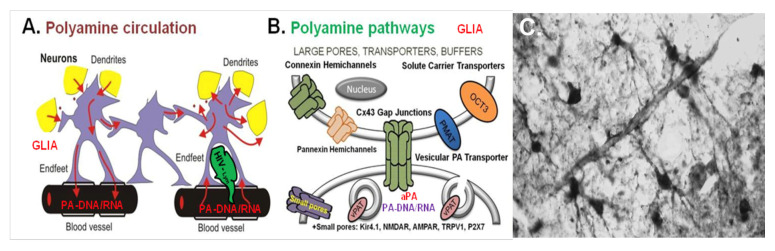
Distinct mechanisms promote circulation of polyamines and acetylated polyamines in the brain. (**A**) Suggested interaction between astrocytes (violet), neuronal dendrites (yellow), synapses (yellow), lymphocytes (green), and blood vessels (black) based on bi-directional polyamine (PA) fluxes (red arrows). PAs and aPAs are taken up and released from glia to neurons as well as propagated distantly through the syncytium. (**B**) Suggested PA and acetylated PAs (aPAs) pathways (uptake and release) in glia via (i) connexin 43 (Cx43) hemichannels (green) or gap-junctions (green), (ii) transporters such as organic cation transporters (OCTs) SLC22A1-3 (orange), and (iii) vesicular PA transporter (vPAT) SLC18B1 with subsequent vesicular uptake/release (brown). Minor pathways (iv) are present in some channels (Kir4.1, NMDAR, AMPAR, TRPV1, P2X7). The scheme represents data from Laube and Veh, 1997; Masuko et al., 2003; Cui et al., 2009; Benedikt et al., 2012; Sala-Rabanal et al., 2013; Merali et al., 2014; Skatchkov et al., 2014; 2015; 2016; Kucheryavykh et al., 2017; Malpica-Nieves et al., 2020; 2021. (**C**) Astrocytes extend their endfeet to small vessels, as shown here after staining of rat hippocampus for spermine-like immunoreactivity.

Transport of PAs in CNS uses neuronal and glial processes (Figure 6) showing multiple and bidirectional PA-fluxes. There is a high-affinity uptake of PAs in rodent cortex [79]. The authors, however, bathed slices of brain tissue in respective solutions and consequently could measure only the uptake from artificial CSF but not from the blood compartment. In living brains, PAs (Figure 4) are accumulated preferentially in glial cells [66,80] but not in neurons [66,73,81,82,83,84]. Unfortunately, the PA transport pathways from neurons to glia, from glia to neurons, and in the astrocytic network are still unknown.

PA transport in the brain has been identified as uptake/release via (i) organic cation transporters (OCT; [72,85,86,87,88,89], (ii) glial gap junctions [90], (iii) connexin-43 hemichannels (Cx43 HCs; [91]), and (iv) minor pathways through PA-permeable receptors and channels [92,93]. PA-transport through ion channels or receptors, however, is negligible when compared to the other fluxes.

### 5.1. Uptake of PAs in Astrocytes via Organic Cation Transporters

Adult astrocytes lack SPD synthesis [68] and use uptake to store PAs, to some degree even in vesicles [94]. Glial cells show several types of PA-uptake [53,72,84,85,86,91]. Recent experimental data [91] make the organic cation transporter 3 (OCT3, SLC22A3) a likely candidate to allow PA uptake in astrocytes. This hypothesis is supported by several lines of evidence. (i) It is present in astrocytes [86,91,95]. (ii) There is a high affinity uptake system in astrocytes with Km-values for SPD/SPM of about 2 µm [85]. As SPD/SPM concentrations in the extracellular space range about 0.5 µm (Table 2), uptake seems possible. Another, so far unidentified, high-affinity uptake system with a Km-value of about 0.5 µm had been described earlier [79] and also would allow PA uptake into astrocytes. (iii) PA uptake into astrocytes is inhibited by trimer 44 NMe, an inhibitor of organic cation transporters [91]. Only OCT3 is expressed in astrocytes in relevant amounts [96]. (iv) OCT3 is rather unspecific and transports quite a number of different compounds [97,98]. This is in good agreement with the fact that in addition to Pas, monoamines, the anesthetic ketamine [98], the anti-Parkinson drug L-Dopa [99], and the anti-diabetic drug metformin [100], as well as SPD can also be transported by a PA transporter into astrocytes [53,72,85,86,91,94,98,99]. PAs can interact with psychoactive substances during transport by OCT3 [98]. The data support a potential role for OCT3 in the mechanism by which astrocytes take up PAs. In strong contrast, however, is the fact that the K_0.5_-value for the uptake of SPM is about 1.0 mM for OCT heterologously expressed in Xenopus oocytes ([88]; see Table 1). At extracellular SPD/SPM concentration of about 0.5 µm (Table 2), which is about 2000-fold lower than the K_0.5_-value, OCT3 cannot provide a relevant contribution to the uptake of PAs by astrocytes. Consequently, the uptake mechanism(s) remain mysterious.

### 5.2. Distribution of PAs via Gap Junctions in the Astroglial Syncytium

Even conceding that the the astroglial network represents no real syncytium, electrical as well as molecular communication between individual astrocytes via gap junctions is intense (Figure 6). Gap junctions, also called electrical synapses, consist of arrays of connexons and macrochannels, which themselves are formed by members of the connexin family proteins. Most connexons are not permeable to PAs, except those formed from connexins Cx38 and Cx43 [20,101,102]. In astrocytes, gap junctions are composed mostly of connexin 43 (Cx43), but lower amounts of Cx26 and Cx30 also are present [103].

Exchange of information in the glial syncytium through these macrochannels is tightly regulated by a variety of extracellular and intracellular factors, including protein kinase C, calcium, and ATP [104]. PAs also belong to these regulators, and SPM at physiological intracellular concentrations (about 1 mM; [105]) efficiently keeps Cx43-containing gap channels open [90] by removing calcium and blocking hydrogen from Cx43 GJs [106,107]. Consequently, PAs may be distributed this way through the astrocytic network.

### 5.3. Release of PAs via Large Pore Connexin-43 (Cx43) Hemichannels

Gap junctions are formed when connexons of one cell dock with another connexon from a neighboring cell [103]. In contrast, when communication with the extracellular matrix is necessary, the cell may use hemi gap junctions (hemichannels, HCs) to exchange molecules with its intercellular environment (Figure 6).

At a resting state, external and internal calcium concentrations ([Ca^+2^]_e_: 1.2 to 1.8 mM; [Ca^+2^]_i_: <100 nM), Cx43 HCs are blocked [108,109,110,111,112,113]. Either strong decrease of ([Ca^+2^]_e_ to about 0.2 mM [110] or mild increase of [Ca^+2^]_i_ to below 500 nm) [113,114], however, relieves this block [115]. On the other hand, PA release may still occur via Cx43 HCs in high [Ca^+2^]_e_ conditions [91] when intracellular calcium is still low or mildly increased.

In case of very strong neuronal activation, Cx43 HCs may be opened because extracellular calcium levels can drop dramatically [116]. During 5 s of spreading depression or 2 min of ischemia, [Ca^+2^]_e_ may decrease to about 0.06 mM [116]. Because this value is below the K_50_ for opening, which is about 0.05–0.2 mM [109,110], Cx43 HCs will be opened. However, neither spreading depression nor ischemia represents physiological situations and need to be considered separately.

In conclusion, opening of HCs with the help of PAs provide pathways for the transfer of small ions, metabolites, and signaling molecules between the cytosol and the extracellular space around astrocytes. Release of PAs through HCs may modulate receptors in neurons such as NMDA and AMPA receptors via the delivery of these PA “gliotransmitters” from their astrocytic store to neurons.

## 6. Catabolism and Interconversion

The catabolism of PAs (Figure 3) in principle follows two pathways. One starts with the acetylation of SPM by cytosolic SPM/SPD-N^1^-acetyltransferase (SSAT), whereas in the second, SPM is directly oxidized via SPM oxidase (SMOX).

### 6.1. The Cetylation Pathway

Apparently, the most important pathway for catabolism and interconversion of PAs (Figure 3) starts with the acetylation of SPM by cytosolic SPM/SPD-N^1^-acetyltransferase (SSAT). N^1^-acetyl-SPM is converted into SPD via peroxisomal N^1^-PA oxidase (PAOX), which is highly specific for acetylated PAs as substrates [69,117,118]. Subsequently, SSAT also acetylates SPD, and the resulting N^1^-acetyl-SPD again is oxidized by PAOX, yielding PUT (Figure 3). SSAT may also further acetylate N^1^-acetyl-SPM to form N^1^,N^12^-diacetyl-SPM [119]. Peroxisomal PAOX then oxidizes N^1^,N^12^-diacetyl-SPM to N^1^-acetyl-SPD, which is converted to PUT, as stated above (Figure 3).

SSAT is the rate-limiting enzyme for the complete pathway [69,120,121]. Consequently, its activity is again intensely controlled to maintain PA homeostasis (Figure 1). This regulation comprises an increased expression of the SAT1 gene and an effective conversion to mRNA at high intracellular PA levels as well as an increased degradation of mRNA and SSAT protein at low intracellular PA levels (for details see [3]). Thus, intracellular PA-homeostasis is obtained by the combined regulation of biosynthetic and degradation pathways (Figure 1).

### 6.2. The Direct Oxidation Pathway

The second catabolic pathway starts with the direct oxidation of SPM via SPM oxidase (SMOX). The emerging aldehyde (N^8^-3-propanal-spermidine, Figure 3) either undergoes a beta-elimination step, yielding SPD and acrolein, or is further oxidized by aldehyde dehydrogenase to the corresponding acid, N^8^-2-carboxyethyl-spermidine [117,122]. SPD then is oxidized by the cytosolic diamine oxidase (DAOX) [117,123] to N^1^-3-propanal-putrescine (Figure 3), which subsequently may be either oxidized to putreanine by aldehyde dehydrogenase or undergo spontaneous beta-elimination to PUT and acrolein [117]. Finally, PUT may be oxidized further via diamine oxidase to form 4-aminobutanal, which is converted to GABA by the cytosolic aldehyde dehydrogenase [117,123]. This alternate pathway for GABA biosynthesis (Figure 3) is known to occur in midbrain dopamine neurons [124] and may be of special importance for the release of GABA by astrocytes [125,126].

Together with biosynthesis, the PA catabolism forms an effective interconversion cycle, enabling mammalian cells to quickly adapt their PA content to the actual demand [117,127]. It must be mentioned, however, that some catabolic reactions produce potentially cytotoxic metabolites like hydrogen peroxide, which might result in DNA damage [69,118]. The intracellular concentration of SPM can reach millimolar levels (Table 2), and thus the degradation of SPM via direct SMOX has been suspected to cause damage to the cell via cytotoxic metabolites [128,129]. Furthermore, a number of authors argue that products of PA oxidation might be involved in neurodegeneration based on the fact that overexpression of enzymes such as PAOX, SMOX and DAOX degrading PAs can result in severe damage of nervous tissue.

With respect to adult, healthy animals, these ideas, however, must be criticized. Most of the reports, which emphasize a high toxicity of PA degradation products, are based on experiments far away from physiological situations. Mostly they depend on the use of tissue culture, where peroxide or aldehydes often are added in grotesque amounts. Actually, experimental evidence for any damage produced by the degradation of PAs under physiological conditions is missing [130]. In addition, PAOX is located in peroxisomes [118,119,131], which allows the oxidation of N^1^-SPM/SPD in a closed environment. The resulting H_2_O_2_ on its own is neither toxic nor able to damage nucleic acids. In the presence of transition metals like copper, however, it may generate toxic hydroxyl radicals in a Fenton-like reaction [132,133]. But peroxisomes actually are free of nucleic acid-associated transition metals like copper. Consequently, the H_2_O_2_ generated within peroxisomes is not toxic and will be disposed of via catalase.

In animal models of injury, however, the situation may be different [134,135,136]. In a very precise report, it was shown that during ischemia, intracerebral levels of 3-aminopropanal are increased, preceding ischemic lesions [136]. Even considering that 3-aminopropanal is a very unstable compound, which spontaneously decays to acrolein and ammonium ions (Figure 3), it may not really be important whether ischemic damage is produced by 3-aminopropanal itself or by acrolein, its decomposition product. So, in pathological situations, PA catabolism actually may contribute to toxic effects in the animal brain.

Catabolism via oxidation may lead to the loss of cellular PAs, since the results of aldehyde dehydrogenase activity (Figure 3), N^8^-2-carboxyethyl-spermidine, putreanine, or GABA are end products of the PA system. The biological advantage of the cytosolic oxidation of SPM via SMOX for the interconversion cycle remains unclear. The two separate pathways, direct versus acetylation-dependent oxidation, may serve different purposes. By speculation, the SSAT pathway may interconvert PAs depending on the cellular demand. This idea is supported by its regulation via PA content (Figure 1). In contrast, the SMOX and the cytosolic pathway may be important to reduce the net PA content inside the cell, independent of fluxes of unmodified or acetylated PAs.

### 6.3. Additional Interconversion Products

In addition to the pathways described above, SPD serves as substrate for deoxyhypusine synthase (Figure 3). The transfer of an aminobutyl group of SPD onto a specific lysine residue is the first step of the posttranslational modification of the factor elF5A. The deoxyhypusine residue is subsequently converted to hypusine by a specific hydroxylase. The translation factor elF5A is essential for early embryonic development [137] and for the activation of the autophagy pathway, and the hypusine modification is crucial for its proper activity [44,138].

Putreanine (Figure 3) is yet another product of PA catabolism [122,139,140]. Initially, putreanine was thought to be a unique metabolite of mammalian central nervous PA catabolism [139] but was later also detected in rat liver and kidney [122]. At present, there is no known biological function for putreanine other than being the terminal product of cytoplasmatic SPD oxidation. Putreanine, however, accumulates in excretory organs, and one may speculate that it simply serves as a carrier for the extracorporal disposal of waste nitrogen. Certainly, to understand the biological role of putreanine more deeply, further investigation is needed.

## 7. Conclusions

In summary, we highlight discrepancies in the literature and add new data on the circulation of PAs in the brain and body, including important sources of PAs in gut lumen, blood plasma, and cerebrospinal fluid. We emphasize a large gap in concentrations between these compartments, resulting in surprisingly high accumulation of PAs in glial cytoplasm. We provide a novel and complete scheme for the biosynthesis/degradation of PAs, including glycine, glutamate, proline and others as PA precursors. We suggest a solution for the apparent contradiction in the literature, suggesting that when ODC knockout seems to be lethal, the AGM pathway is probably rescuing PUT production. We point to PA transport in glial cells, explaining how without synthesis these cells (but not neurons) preferentially accumulate PAs. We introduce a novel and complete scheme for PA interconversion, including hypusine, putreanine, and the unique gliotransmitter (GABA) as end-products. In addition, we highlight that S-adenosylmethionine decarboxylase promotes decreased PUT and increased SPM levels. This review can serve as an updated contribution to understanding the PA mystery in the CNS and its body.

## Figures and Tables

**Table 1:** Designations, structures, and biological or pharmacological functions of important polyamines.

Source	Substance	Sum Formula	Mass/g*mol^−1^	Structure	Function/Purpose/Usage	References
plants/protozoa	Diaminopropane	C_3_H_10_N_2_	74,13	NH_2_(CH_2_)_3_NH_2_	proliferative agent	(1)
ubiquitous	Putrescine	C_4_H_12_N_2_	88,15	NH_2_(CH_2_)_4_NH_2_	precursor to spermidine	(2)
prokaryotes/E.coli	Cadaverine	C_5_H_14_N_2_	102,18	NH_2_(CH_2_)_5_NH_2_	decarboxylation product of L-Lysine	(3)
eukaryotes	Norspermidine	C_6_H_17_N_3_	131,22	NH_2_(CH_2_)_3_NH(CH_2_)_3_NH_2_	catabolic metabolite	(4)
ubiquitous	Spermidine	C_7_H_19_N_3_	145,25	NH_2_(CH_2_)_3_NH(CH_2_)_4_NH_2_	growth regulator in eukaryotic cells	(5)
ubiquitous	N1-Acetylspermidine	C_9_H_21_N_3_O	187,28	CH_3_CONH(CH_2_)_3_NH(CH_2_)_4_NH_2_	catabolic metabolite	(6)
prokaryotes/E.coli	Aminopropylcadaverine	C_8_H_21_N_3_	159,27	NH_2_(CH_2_)_5_NH(CH_2_)_3_NH_2_	compensatory metabolite/growth regulator	(7)
plants/prokaryotes/algae	Homospermidine	C_8_H_21_N_3_	159,27	NH_2_(CH_2_)_4_NH(CH_2_)_4_NH_2_	essential precursor to pyrrolizidine alkaloids	(8)
plants/prokaryotes/algae	Norspermine	C_9_H_24_N_4_	188,31	NH_2_(CH_2_)_3_NH(CH_2_)_3_NH(CH_2_)_3_NH_2_	antiproliferative agent	(9)
plants/prokaryotes/algae	Thermospermine	C_10_H_26_N_4_	202,34	NH_2_(CH_2_)_3_NH(CH_2_)_3_NH(CH_2_)_4_NH_2_	growth regulator in plants	(10)
eukaryotes/prokaryotes	Spermine	C_10_H_26_N_4_	202,34	NH_2_(CH_2_)_3_NH(CH_2_)_4_NH(CH_2_)_3_NH_2_	growth regulator in eukaryotic cells	(5)
eukaryotes	N1-Acetylspermine	C_12_H_28_N_4_O	244,38	CH_3_CONH(CH_2_)_3_NH(CH_2_)_4_NH(CH_2_)_3_NH_2_	catabolic metabolite	(6)
prokaryotes/E.coli	Bisaminopropylcadaverine	C_11_H_28_N_4_	216,37	NH_2_(CH_2_)_3_NH(CH_2_)_5_NH(CH_2_)_3_NH_2_	compensatory metabolite/growth regulator	(7)
plants/fungi	Canavalmine	C_11_H_28_N_4_	216,37	NH_2_(CH_2_)_4_NH(CH_2_)_3_NH(CH_2_)_4_NH_2_	growth inhibitor in murine leukemia cells	(11)
prokaryotes/E.coli	Homospermine	C_12_H_30_N_4_	230,39	NH_2_(CH_2_)_4_NH(CH_2_)_4_NH(CH_2_)_4_NH_2_	growth regulator in root nodule bacteria	(12)
thermophiles	Caldopentamine	C_12_H_31_N_5_	245,41	NH_2_(CH_2_)_3_NH(CH_2_)_3_NH(CH_2_)_3_N(CH_2_)_3_NH_2_	survival at extreme temperature	(13)
prokaryotes/E.coli	Aminopropylcanavalmine	C_14_H_35_N_5_	273,46	NH_2_(CH_2_)_3_NH(CH_2_)_4_NH(CH_2_)_3_N(CH_2_)_4_NH_2_	compensatory metabolite/growth regulator	(7)
plants	Homopentamine	C_16_H_39_N_5_	301,51	NH_2_(CH_2_)_4_NH(CH_2_)_4_NH(CH_2_)_4_N(CH_2_)_4_NH_2_	growth/differentiation	(14)
thermophiles	Caldohexamine	C_15_H_38_N_6_	302,5	NH_2_(CH_2_)_3_NH(CH_2_)_3_NH(CH_2_)_3_NH(CH_2_)_3_NH(CH_2_)_3_NH_2_	inhibition of PA-uptake	(9)
thermophiles	Homocaldohexamine	C_16_H_40_N_6_	316,53	NH_2_(CH_2_)_3_NH(CH_2_)_3_NH(CH_2_)_3_NH(CH_2_)_3_NH(CH_2_)_4_NH_2_	antiviral agent in plants	(15)
prokaryotes	Thermohexamine	C_16_H_40_N_6_	316,53	NH_2_(CH_2_)_3_NH(CH_2_)_3_NH(CH_2_)_4_NH(CH_2_)_3_NH(CH_2_)_3_NH_2_	inhibition of PA-uptake	(9)
plants/mammals	Agmatine	C_5_H_14_N_4_	130,19	[(NH_2_)CNH]NH(CH_2_)_4_NH_2_	neurotransmitter/precursor to putrescine	(16)
plants	N6-Methylagmatine	C_6_H_16_N_4_	144,22	[(NH_2_)CN(CH_3_)]NH(CH_2_)_4_NH_2_	nutrient	(17)
PA-analogue	Methylglyoxalbisguanylhydrazone (MGBG)	C_5_H_12_N_8_	184,2	(NH_2_)(NH)CNHNCHC(CH_3_)NNHC(NH)(NH_2_)	antileucamic agent	(18)
PA-analogue	MDL 27695	C_27_H_44_N_4_	424,7	C_6_H_5_CH_2_NH_2_(CH_2_)_3_NH(CH_2_)_7_NH(CH_2_)_3_NH_2_CH_2_C_6_H_5_	antimalaria agent	(19)
PA-analogue	N1,N11-Bisethylnorspermine	C_13_H_32_N_4_	244,42	C_2_H_5_NH_2_(CH_2_)_3_NH(CH_2_)_3_NH(CH_2_)_3_NH_2_C_2_H_5_	antiproliferative agent	(20)
PA-analogue	BE 4-4-4-4	C_20_H_47_N_5_	357,6	NH_2_(CH_2_)_4_NH(CH_2_)_4_NH(CH_2_)_4_N(CH_2_)_4_NH_2_	antiproliferative agent	(21)
PA-analogue	trimer 44NMe	C_33_H_69_N_9_	592	[1,3,4][(CH_2_)NH(CH_2_)_4_NH(CH_2_)_4_NH_2_]_3_(C_6_H_6_)	antiproliferative agent	(22)
Streptomyces spp.	Kanamycin A	C_18_H_40_N_4_O_11_	488,5	6-*O*-(3-Amino-3-desoxy-α-d-glucopyranosyl)-4-*O*- (6-amino-6-desoxy-α-d-glucopyranosyl)-2-desoxy- d-streptamin	aminoglycoside antibiotic agent	(23)
Streptomyces spp.	Neomycin B	C_23_H_46_N_6_O_13_	614,6	4-*O*-2,6-Diamino-2,6-didesoxy-α-d-glucopyranosyl-5-*O*- [3-*O*-2,6-diamino-2,6-dideoxy-β-l-idopyranosyl- β-d-ribofuranosyl]-2-deosxy-d-streptamin	aminoglycoside antibiotic agent	(23)
**References**						
(1) Potter, M.J.; Gibson, M.K.; McCammon, J.A.; *J. Am. Chem. Soc.* **1994**, *116*, 10298–10299.						
(2) Takao, K.; Sugita, Y.; Shirahata, A. *Amino Acids* **2010**, *38*, 533–539						
(3) Li, M.; Li, D; Huang, Y.; Liu, M.; Wang, H.; Tang, Q.; Lu, F. *J. Ind. Microbiol. Biotechnol.* **2014**, *41*, 701–709						
(4) Michael, A.J. *J. Biol. Chem.* **2016**, *291*, 14896–14903						
(5) Bergeron, R.J.;McManis, J.S.; Weimar, W.R.; Schreier, K.; Gao, F.; Wu, Q.; Ortiz-Ocasio, J.; Luchetta, G.R.; Porter, C.; Vinson, J.R.T. *J. Med. Chem.* **1995**, *38*, 2278–2285						
(6) Yu, C.; Liu, R.; Xie, C.; Zhang, Q.; Yin, Y.; Bi, K.; Li, Q. *Anal. Bioanal. Chem.* **2015**, *407*, 6891–6897						
(7) Igarashi, K.; Kashiwagi, K.; Hamasaki, H.; Miura, A.; Kakegawa, T.; Hirose, S.; Matsuzaki, S. *J. Bacteriol.* **1986**, *166*, 128–134						
(8) Ober, D.; Gibas, L.; Witte, L.; Hartmann, T. *Phytochemistry* **2003**, *62*, 339–344						
(9) Takao, K.; Sugita, Y.; Shirahata, A. *Amino Acids* **2010**, *38*, 533–539						
(10) Takano, A.; Kakehi, J.-I.; Takahashi, T. *Plant Cell Physiol.* **2012**, *53*, 606–616						
(11) Fujihara, S.; Nakashima, T.; Kurogochi, Y. *Biochem. Biophys. Acta* **1984**, *805*, 277–284						
(12) Fujihara, S.; Harada, Y. *Biochem. Biophys. Res. Commun.* **1989**, *165*, 659–666						
(13) Oshima T.; Moriya T.; Terui Y. *Polyamines. Methods in Molecular Biology (Methods and Protocols)*; Pegg, A., Casero, R., Jr., Eds.; Humana Press, 2011; Volume 720, pp. 81–111						
(14) Bagni, N.; Tassoni, A. *Amino Acids* **2001**, *20*, 301–317						
(15) Sagor, G.H.M.; Liu, T.; Takahashi, H.; Niitsu, M.; Berberich, T.; Kusano, T. *Plant Cell Rep.* **2013**, *32*, 1477–1488						
(16) Weiss, T.; Bernard, R.; Bernstein, H.-G.; Veh, R.W.; Laube, G. *Transl. Psychiatry* **2018**, *8*, 201						
(17) Paik, W.K.; Kim, S. *Amino Acids* **1993**, *4*, 267–286						
(18) Von Hoff, D.D. *Ann. Oncol.* **1994**, *5*, 487–493						
(19) Edwards, M.L.; Stemerick, D.M.; Bitonti, A.J.; Dumont, J.A.; McCann, P.P.; Bey, P.; Sjoerdsma, A. *J. Med. Chem.* **1991**, *34*, 569–574						
(20) Thomas, T.J.; Thomas, T. *Med. Sci.* **2018**, *6*, 24						
(21) Basu, H.S.; Pellarin, M., Feuerstein, B.G.; Shirahata, A.; Samejima, K.; Deen, D.F.; Marton, L.J. *Cancer Res.* **1993**, *53*, 3948–3955						
(22) Muth, A.; Madan, M.; Archer, J.J.; Ocampo, N.; Rodriguez, L.; Otto Phanstiel, O. *J. Med. Chem.* **2014**, *57*, 348−363						
(23) Blagbrough I.S.; Metwally A.A.; Andrew J. Geall A.J. *Polyamines. Methods in Molecular Biology (Methods and Protocols)*; Pegg A., Casero, R., Jr., Eds.; Humana Press, 2011; Volume 720, pp. 493–503						

**Table 2:** Polyamine concentrations in brain and body fluids.

	Arg		PUT	NAc-PUT	ref.		SPD	NAc-SPD	ref.		SPM	NAc-SPM	Reference
serum	80 µM		100 nM		(4)		130 nM		(4)		40 nM		(4)
			130 nM		(3)		400 nM		(3)		50 nM		(3)
							320 nM		(1)		35 nM		(5)
			60 nM	2.5 nM	(8)		4.0 nM		(8)		43 nM		(8)
cerebrospinal fluid			180 nM		(2)		150 nM		(2)		90 nM		(2)
			230 nM		(6)		120 nM		(6)		140 nM		(6)
brain extracellular space			750 nM		(5)		420 nM		(5)		480 nM		(5)
cytoplasm (fibroblasts)			29 µM		(4)		159 µM		(4)		635 µM		(4)
cytoplasm (ascites cells)			43 µM		(4)		430 µM		(4)		602 µM		(4)
hepatocytes							1150 µM		(9)		880 µM		(9)
brain (Müller cells)											800 µM		(7)
urine			60.2 nM	2.5 nM	(8)		4.0	1.7 nM	(8)		43.1 nM	1.3 nM	(8)
daily loss			90.3 nmoles	3.7 nmoles	(8)		6.0 nmoles	2.5 nmoles	(8)		64.6 nmoles	1.9 nmoles	(8)
**References**													
(1)	Marton, L.J.; Russell, D.H.; Levy, C.C. *Clin. Chem.* **1973**, *9*, 923–926												
(2)	Marton, L.J.; Heby, O.; Levin, V.A.; Lubich, W.P.; Crafts, D.C.; Wilson, C.B. *Cancer Res.* **1976**, *36*, 973–977												
(3)	Bartos, F.; Bartos, D.; Grettie, D.P.; Campbell, R.A. *Biochem. Biophys. Res. Commun.* **1977**, *75*, 4												
	Seiler, N.; Atanassov, C.L. *Progr. Drug Res.* **1994**, *43*, 87–141												
(4)	Morgan, D.M.L. *Biochem. Soc. Trans.* **1990**, *18*, 1080–1084												
(5)	Dot, J.; Lluch, M.; Blanco, I.; Rodríguez-Alvarez, J. *J. Neurochem.* **2000**, *75*, 1917–1926												
	Dot, J.; Danchev, N.; Blanco, I.; Rodríguez-Alvarez, J. *Neuroreport* **2002**, *13*, 1083–108												
(6)	Ekegrena, T.; Gomes-Trolin, C. *Anal. Biochem.* **2005**, *338*, 179–185												
(7)	Kucheryavykh, Y.V.; Shuba, Y.M.; Antonov, S.M.; Inyushin, M.Y.; Cubano, L.; Pearson, W.L.; Kurata, H.; Reichenbach, A.; Veh, R.W.; Nichols, C.G.; Eaton, M.J.; Skatchkov, S.N. *Glia* **2008**, *56*, 775–790												
(8)	Liu, R.; Li, Q.; Ma, R.; Lin, X.; Xu, H.; Bi, K. *Anal. Chim. Acta* **2013**, *791*, 36–45												
(9)	Igarashi, K.; Kashiwagi, K. *Int. J. Biochem. Cell Biol.* **2019**, *107*, 104–115												

## Data Availability

Additional data put in the Supplement section and the raw data supporting the conclusions of this review will be made available without undue reservation.

## Supplementary Materials

The following supporting information can be downloaded at: https://www.mdpi.com/article/10.3390/biom12040501/s1, Figure S1: Photographic documentation.

## Abbreviations

CNS	central nervous system
CSF	cerebrospinal fluid
PA	polyamine
PUT	putrescine
SPD	spermidine
SPM	spermine
AGM	agmatine
ODC	ornithine decarboxylase
dcAdoMet	decarboxylated S-adenosylmethionine
AdoMetDC	S-adenosylmethionine decarboxylase
SSAT	spermidine/spermine acetyltransferase
SMOX	spermine oxidase
PAOX	polyamine oxidase
DAOX	diamine oxidase
OCT3	organic cation transporter 3
SLC22A3	solute carrier protein 22A3 (organic cation transporter 3)
Cx	connexin
HC	hemichannel
GABA	gamma aminobutyrate
ATP	adenosine triphosphate
CAT1	cationic amino acid transporter 1
PKC	protein kinase C
NMDAR	N-methyl-d-aspartate-receptor
AMPAR	α-amino-3-hydroxy-5-methyl-4-isoxazolepropionic acid receptor
