# Supplementary material for: Unique Chemistry, Intake, and Metabolism of Polyamines in the Central Nervous System (CNS) and Its Body
Source: Biomolecules. 2022 Mar 25;12(4):501. doi: 10.3390/biom12040501 (PMC9025450; doi:10.3390/biom12040501)
Supplement: Supplementary file 1 [file biomolecules-12-00501-s001.zip › biomolecules-1551577-supplementary.pdf]

Supplement

Methods Section for Figures 4 to 6 and the Supplementary Figure

Animals

All animal experiments were approved by the Regional Berlin Animals Ethics Committee and conducted in strict accordance with the European Communities Council directive regarding care and use of animals for experimental procedures. Adult male Wistar rats, weighing 250-300 g were obtained from an institutional breeder (Department for Experimental Medicine (FEM), Charité University Medicine Berlin). Animals were group-housed under controlled temperature (22 °C) and illumination (12 hour cycle) with water and food ad libitum. A total of 46 animals was analyzed in the present report.

Perfusion fixation

Rats were deeply anaesthetized by intraperitoneal injections of a cocktail consisting of 45 % ketamine (100 mg⁄ml; Ketavet), 35% xylazine (20 mg⁄ml; Rompun) and 20 % saline, at a dose of 0.16 ml⁄100 g of body weight, supplemented by 200 IU heparin i. p. to avoid clocking of brain vessels during surgery. Subsequently, animals were fixed via transcardial perfusion with PGPic (4 % paraformaldehyde, 0.05 % glutaraldehyde, and 0.2 % picric acid in 0.1 M phosphate buffer, pH 7.4 (Somogyi and Tagaki, 1982). Brains were removed, cryoprotected in 0.4 M sucrose for about 4 h and in 0.8 M sucrose overnight, cut into blocks at preselected rostro-caudal levels, shock-frozen in hexane at -70 °C, and stored at -80 °C until use.

Antibodies

Primary antibodies were raised in the author´s lab (rabbit anti-SPD/SPM, rabbit anti-ornithine) as described ago [66]. The same technique was used to produce rabbit anti-N-

acetylspermine antibodies (see Supplemental Figure) and the rabbit anti-acrolein antibody was obtained fom MoBiTec, Göttingen, Germany (rabbit anti-acrolein; LS-C63521). Biotin labeled secondary antibodies and Elite ABC complex were from Vector (Vector Laboratories, Burlingame, CA, USA).

Immunocytochemistry

Freely floating serial brain cryostat sections (25 µm) were subjected to immunocytochemistry as described earlier (Bernard and Veh, 2012). In short, sections were rinsed in PBS (phosphate buffered saline; 150 mM sodium chloride in 10 mM phosphate buffer, pH 7.4), treated for 15 min with 1% sodium borohydride in PBS to remove residual aldehyde groups from the fixative, and again thoroughly washed in PBS. Sections were pretreated for 30 minutes in a blocking and permeabilizing solution (10% normal goat serum in 0.3% Triton X-100 and 0.05% phenylhydrazine in PBS at room temperature (RT).

Primary antibodies (see above) were applied for 36 hours at appropriate dilutions in PBS containing 10% NGS, 0.3% Triton X-100, 0.1% sodium azide, and 0.01% thimerosal at 4° C. Sections were thoroughly rinsed in PBS, pretreated for 1 hour with PBS-A (0.2% bovine serum albumine in PBS), and exposed for another 24 hours at RT to the secondary antibody, diluted 1:2.000 in PBS-A containing 0.3% Triton X-100 and 0.1% sodium azide.

After repeated washings in PBS and preincubation for one hour in PBS-A, the Elite avidin-biotin-peroxidase-complex (1:200 dilution in PBS-A) was attached to biotinylated secondary antibodies for another 12 hours at RT.

After additional rinses in PBS, preincubation for 15 minutes in a solution of 0.5 mg/ml diaminobenzidine, 3 mg/ml ammonium nickel sulfate, and 10 mM imidazole in 50 mM Tris buffer, pH 7.6, the visualization of the antigen-antibody complexes was started by the addition of 0.0015% hydrogen peroxide and stopped after 15 minutes at RT by repeated

washings with PBS. Sections were mounted onto gelatine-coated slides, air-dried not longer than 30 minutes, dehydrated through a graded series of dilutions of ethanol, transferred into xylene, and coverslipped with Entellan.

Figure S1

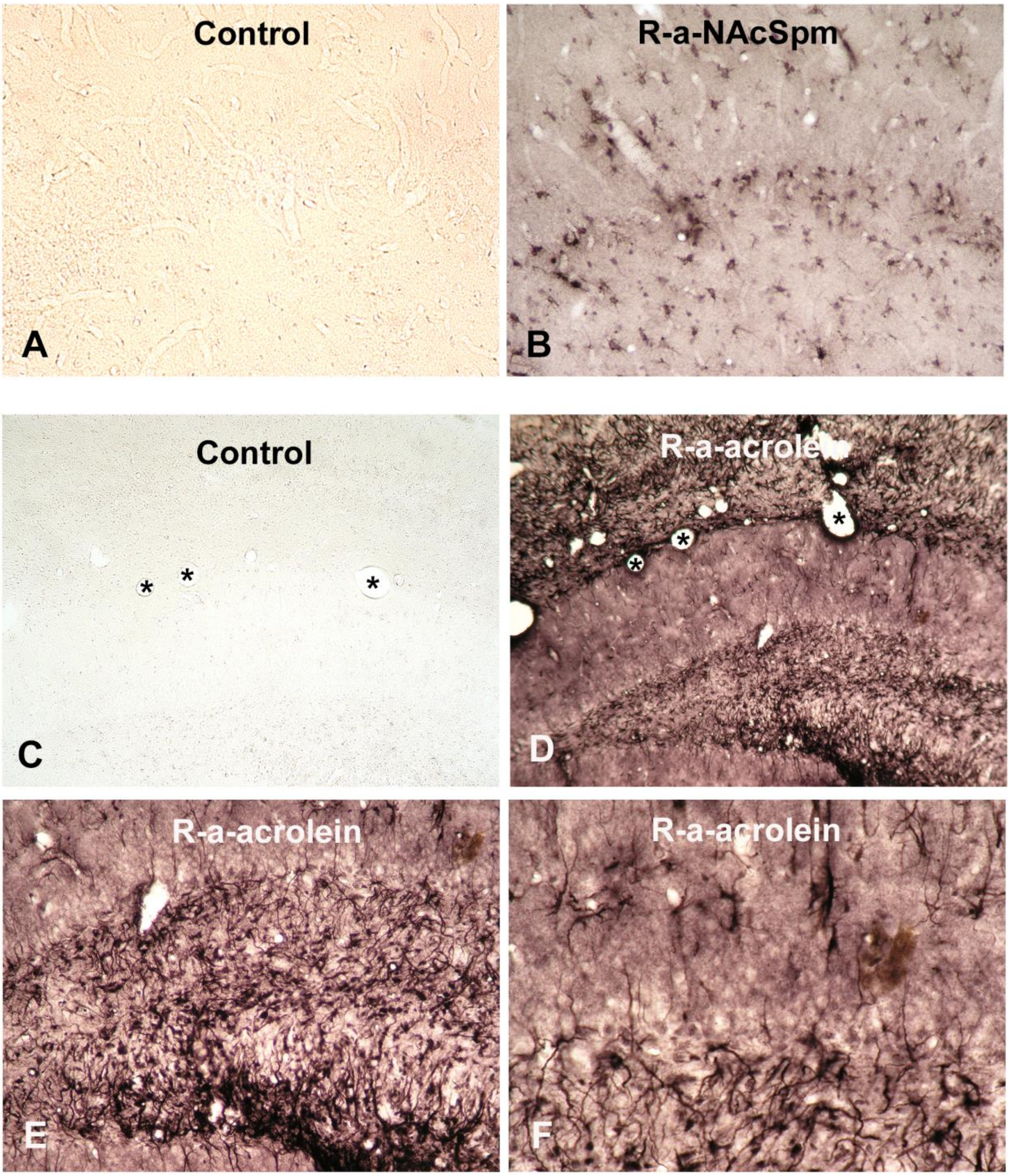

Supplementary Figure

Photographic documentation

Microscopic images were obtained with upright Leica DMRB light microscope connected to a high-resolution digital camera (Olympus SP-55UZ). Primary photographs were adjusted for brightness, color, and contrast in Adobe Photoshop CS3 (10.0) and arranged using Adobe Illustrator CS3 (13.0.0).

Legend to Supplementary Figure S1

Immunocytochemical visualization of anti-N-acetylspermine and anti-acrolein staining in the hippocampus of epileptic (pilocarpin-treated) rats (data from Höhlig et al, this special issue). Horizontal sections treated with the anti-N-acetylspermine antibody (B) show strongly stained astrocytes in the molecular layer and especially in the hilus (bottom half) of the dentate gyrus. The control section (A) remains blank.

Frontal sections through the brain of another epileptic animal display strong anti-acrolein immunoreactivity (D to F) in the hippocampus, while the control section (C) remains empty. Note the identical positions of capillaries (asterisks) in the closely spaced sections (C) and (D). At higher magnifications the strong staining of individual astrocytes becomes even more evident.
